# MiR-146a induction by cyanobacterial lipopolysaccharide antagonist (CyP) mediates endotoxin cross-tolerance

**DOI:** 10.1038/s41598-018-29820-w

**Published:** 2018-07-27

**Authors:** Monica Molteni, Annalisa Bosi, Vincenzo Saturni, Carlo Rossetti

**Affiliations:** 1 Dipartimento di Biotecnologie e Scienze della Vita, Università degli studi dell’Insubria, Via Dunant, 3 - 21100 Varese, Italy; 2 Servizio di Immunoematologia e Medicina Trasfusionale - Ospedale di Circolo Fondazione Macchi - ASST Settelaghi, Viale Borri, 57- 21100 Varese, Italy

## Abstract

Endotoxin tolerance is a phenomenon characterized by a reduced capacity of monocytes and macrophages to respond to repeated stimulation with lipopolysaccharide (LPS) which has been suggested to represent a way of controlling the intensity and duration of innate immune response. During endotoxin tolerance, monocytes undergo functional re-programming primarily by epigenetic regulation. Recently, micro-RNA (miR)-146a has been demonstrated to be the major player of the negative regulation of the pro-inflammatory response, affecting TNF-α production. In this study, we have employed CyP, a cyanobacterial LPS antagonist acting on TLR4-MD2 complex, for priming human monocytes and evaluating their response to a subsequent challenge with *E*. *coli* LPS. Results show that CyP is able to induce cross-tolerance to *E*. *coli* LPS by inhibiting TNF-α production. The mechanism of action is mediated by a specific induction of miR-146a and reduction of IRAK1 and TRAF6 expressions in human monocytes by CyP priming. Up-regulation of miR-146a by CyP alone, affects subsequent cell response in term of TNF-α production even when monocytes are incubated with other TLR ligands, as lipoteichoic acid (LTA), thus confirming miR-146a as a critical player mediating TNF-α regulation during cross-tolerance with CyP.

## Introduction

Lipopolysaccharide (LPS) is the main component of the outer membrane of gram-negative bacteria able to elicit a strong activation of innate immune cells, through interaction with Toll-Like Receptor (TLR) 4. LPS challenges, *in vitro* and *in vivo*, induce the release of high amounts of pro-inflammatory mediators, such as TNF-α, IL-1β and IL-6 by polymorphonuclear cells, monocytes and macrophages. The response is tightly controlled; the triggering of pro-inflammatory response with low concentrations of LPS is known to induce a transient refractory state in cells, so that, cells display a less robust induction of pro-inflammatory mediators at a second challenge with a higher dose of LPS. This phenomenon, known as endotoxin tolerance, is thought to be evolved in order to prevent excessive harmful stimulation due to the continuous exposure to the same stimulus. *In vivo*, the priming of mice, obtained by injecting a sublethal dose of LPS, protects them from a subsequent lethal dose of LPS^1^. In healthy human individuals treated with low repeated intravenous doses of LPS, circulating monocytes show a tolerant phenotype, characterized by reductions of pro-inflammatory cytokines, TNF-α and IL-6, and anti-inflammatory cytokines (IL-10 and IL1-RA)^2–4^. Endotoxin tolerance has also been extensively studied *in vitro*, in monocytes, macrophages, monocytic cell lines, showing a downregulation of genes coding for many pro-inflammatory cytokines and chemokines^5,6^, and an up-regulation of several other genes coding for anti-inflammatory cytokines, chemokines, and antimicrobial products^7,8^. These results outline the profound gene reprogramming occurring in monocytes and macrophages during the induction of tolerance, a phenomenon that has been shown to be widely dependent on specific epigenetic regulations^9–11^. Differently from adaptive immunological memory, in which B and T cell responses are characterized by gene rearrangements after antigen re-challenge, innate immune cells can adapt their response after previous insults by modifying their transcription programs without involving permanent genetic changes. Likewise to other mechanisms of innate immune memory or “trained immunity”, endotoxin tolerance has been demonstrated to be associated to histone modifications with chromatin reconfiguration and micro-RNA (miRNA) induction^12–14^. In particular, miR-146a has been found to be the master regulator of TNF-α production in monocytic THP-1 cell-based endotoxin tolerance. Nahid and colleagues have recently demonstrated that miR-146a alone is able to induce LPS tolerance via specific negative regulation of IRAK1 (IL-1 receptor-associated kinase 1) and TRAF6 (TNF receptor-associated factor 6) adaptor proteins, resulting in inhibition of NF-κB activation^15^.

Cyanobacterial product (CyP) is structurally a lipopolysaccharide isolated from the freshwater cyanobacterium *Oscillatoria planktothrix sp*. *FP1* showing potent LPS antagonist activity^16,17^. CyP is characterized by an outer shield of rhamnose, in the O-antigen region, and mainly by galacturonic acid in the inner core, linked to a glucosamine disaccharide backbone bearing hydroxylated and non-hydroxylated fatty acid residues. Compared with gram-negative LPS, CyP lacks 3-deoxy-D-mannooctulosonic acid (KDO), heptose and phosphates^18,19^. It has been demonstrated that CyP is able to down-regulate the pro-inflammatory response activated either by gram-negative LPS or by TLR4 endogenous ligands, by targeting the TLR4-MD2 receptor complex^16,20,21^; CyP is shown to specifically target MD2, thus interfering with the binding of the agonists at the receptor complex and blocking the activation of the pro-inflammatory cascade. Indeed, inhibition of LPS-induced TNF-α production by CyP has been demonstrated to be mediated by an effect on TNF-α mRNA stability^16^, indicating that CyP antagonist action may involve post-transcriptional regulations. Based on this observation, and on several studies demonstrating the role of miRNA and, particularly of miR-146a, in TNF-α regulation during endotoxin tolerance, we have employed CyP in experiments of cross-tolerance with the aim of studying whether CyP pretreatment could affect subsequent monocyte response to LPS triggering, and if miRNA may be implied in this phenomenon. Results demonstrate that CyP priming significantly influences cytokine response to subsequent LPS triggering in THP-1 monocytic cell line and human monocytes; the regulation is only partly similar to that exerted by LPS priming in endotoxin homogeneous tolerance. Similarly to LPS priming, CyP pretreatment induces a state of cell refractoriness to further LPS stimulation in term of TNF-α production, which is shown to be dependent on miR-146a induction and inhibition of IRAK1 and TRAF6 expression.

## Results

### CyP induces cross-tolerance to *E*. *coli* LPS in THP-1 cells and human monocytes

**Figure 1:**
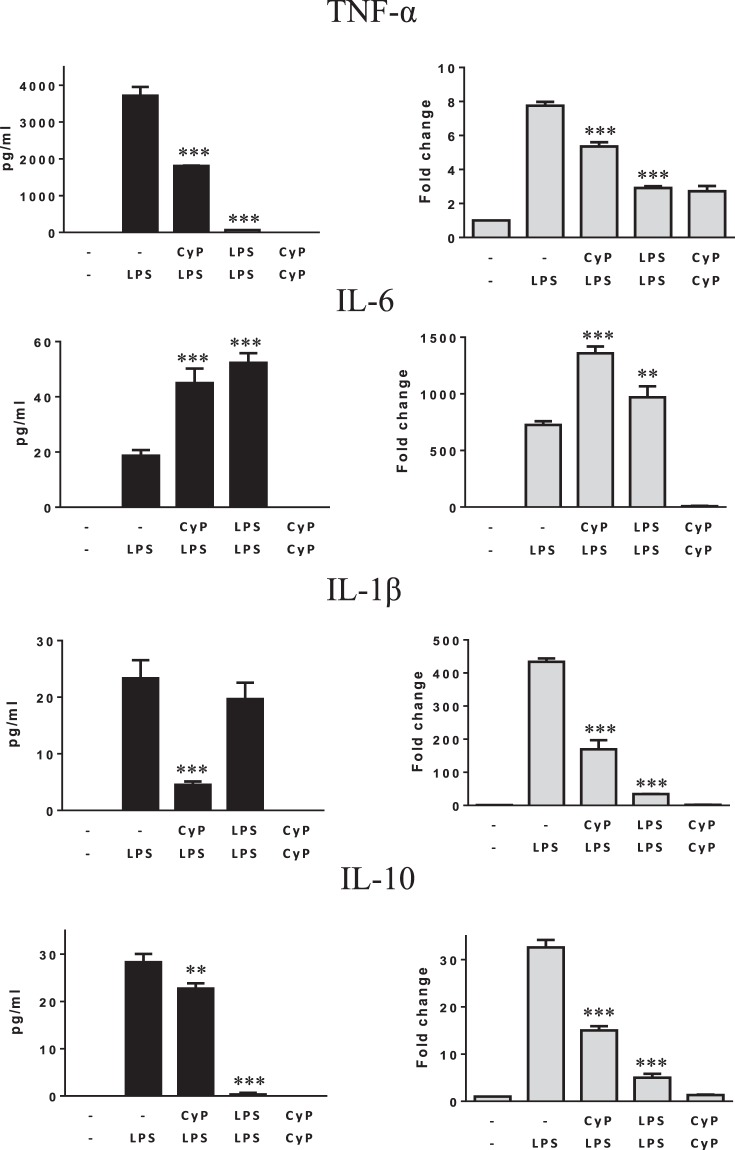
Effects of CyP priming in experiments of cross-tolerance to *E*. *coli* LPS in THP-1 cells. THP-1 cells were preincubated for 16–18 h with CyP (10 µg/ml) for cross-tolerance induction or *E*. *coli* LPS (1 µg/ml) for homogeneous tolerance induction, then washed twice and incubated with *E*. *coli* LPS (1 µg/ml) for 5 h. Untreated THP-1 cells (−/−) were maintained in standard medium during the course of the experiment, whereas LPS-activated cells (−/LPS) were maintained in standard medium during the priming phase and then challenged with *E*. *coli* LPS (1 µg/ml). Cells incubated twice with CyP (10 µg/ml) were also included in each experimental setting. TNF-α, IL-6, IL-1β, IL-10 were measured in cell culture supernatants by ELISA; mRNA expression was analyzed by RT-PCR and given as fold change over the mRNA level expressed by untreated cells (−/−). The results are shown as mean ± S.D. of triplicate cultures, **p < 0,01; ***p < 0,001 (ANOVA with Tukey’s multiple comparison post hoc test).

Preliminary experiments to evaluate the effects of CyP pretreatment on cytokine produced after LPS challenge were performed on THP-1 monocytic cell line (Fig. 1). Cells were primed with CyP 10 µg/ml for 16–18 h, washed twice with PBS, and then challenged with *E*. *coli* LPS 1 µg/ml for 5 h. The time of priming was chosen, based on the results of Nahid *et al*.^15,22^. Untreated THP-1 cells (−/−) were maintained in standard medium during the entire experimental time, whereas LPS-activated cells (−/LPS) were maintained in standard medium during the priming step, washed twice and then challenged with LPS (1 µg/ml). As controls, cells challenged twice with *E*. *coli* LPS (1 µg/ml) for tolerance induction (homogenous tolerance), and cells treated twice with CyP were analyzed. Pro-inflammatory cytokines TNF-α, IL-6 and IL-1β, which are well known to be involved in LPS tolerance induction^5,7,8^, were measured in cell culture supernatants and cytokine mRNA expressions were evaluated after RNA extraction. Results showed that CyP priming induced a significant down-regulation of TNF-α and an increasing of IL-6 productions evaluated at the level of cytokines released in cell culture supernatants as well as at the level of mRNA expressions in comparison with LPS-activated cells (Fig. 1). Although CyP is a LPS antagonist, the effects on TNF-α and IL-6 were similar to those observed in LPS-tolerant cells. The production of IL-1β was low in LPS-activated THP-1 cells; indeed, CyP priming almost completely inhibited production and mRNA expression of IL-1β (Fig. 1).

The homogenous tolerance induction has been demonstrated to involve also the regulation of anti-inflammatory cytokine and chemokine expressions^8,23^. The effects of CyP pretreatment on the production of IL-10 were evaluated in THP-1 cells. Results, shown in Fig. 1, demonstrated that THP-1 cells released low levels of IL-10 in cell culture supernatants after LPS single dose challenge; indeed, CyP priming was effective in down-regulating IL-10 production and mRNA expression, although the effect was less evident compared with that observed in LPS-tolerant cells.

**Figure 2:**
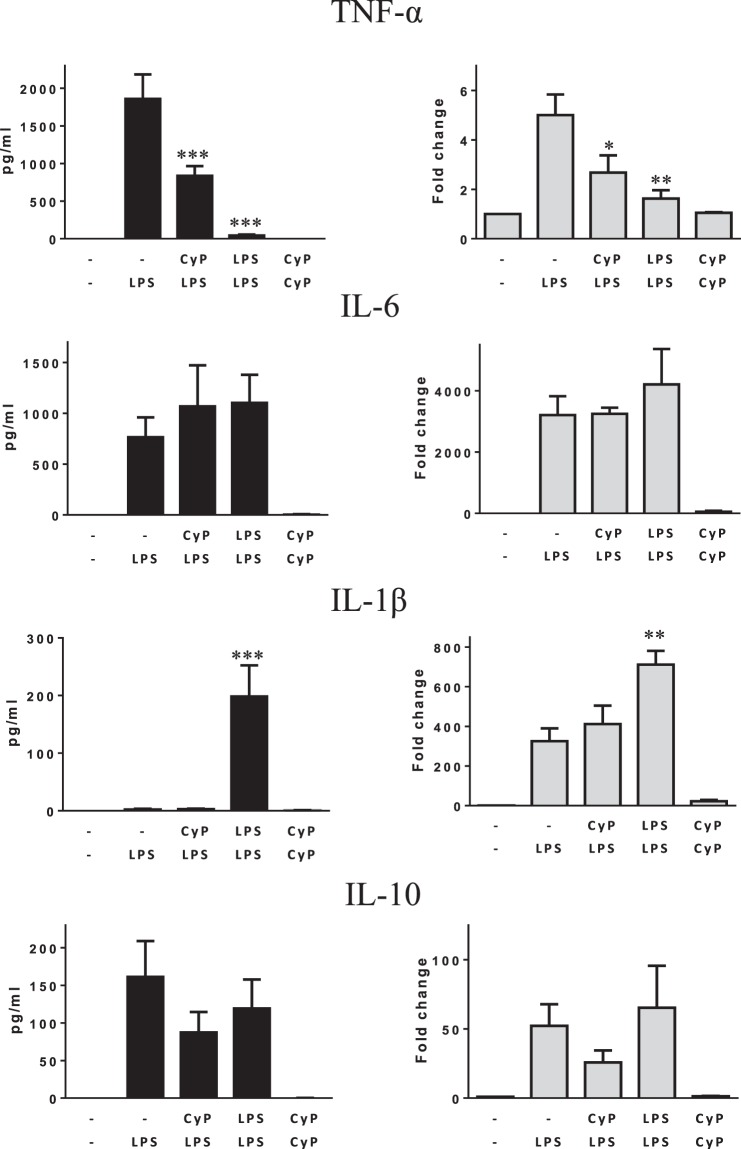
Effects of CyP priming in experiments of cross-tolerance to *E*. *coli* LPS in human primary monocytes. Human primary monocytes isolated by immunomagnetic beads were preincubated with CyP (10 µg/ml) for cross-tolerance induction or *E*. *coli* LPS (1 µg/ml) for homogeneous tolerance induction, then washed twice and incubated with *E*. *coli* LPS (1 µg/ml) for 5 h. Untreated monocytes (−/−) were maintained in standard medium during the course of the experiment, whereas LPS-activated cells (−/LPS) were maintained in standard medium during the priming phase and then challenged with *E*. *coli* LPS (1 µg/ml). Cells incubated twice with CyP (10 µg/ml) were also included in each experimental setting. TNF-α, IL-6, IL-1β, IL-10 were measured in cell culture supernatants by ELISA; mRNA expression was analyzed by RT-PCR and given as fold change over the mRNA level expressed by untreated cells (−/−). The results are shown as mean ± S.E. of at least six independent experiments for cytokines in the supernatants and four independent experiments for mRNA expression *p < 0,05; **p < 0,01; ***p < 0,001 (ANOVA with Tukey’s multiple comparison post hoc test).

Further experiments were done on monocytes from healthy human volunteers to study whether the effects of CyP priming observed on THP-1 could be confirmed (Fig. 2). Even though a certain inter-individual variability in cell response was observed, results on TNF-α confirmed a significant inhibition of both TNF-α production in cell culture supernatants and mRNA expression by CyP priming, thus suggesting that CyP effectively induces cross-tolerance at the level of TNF-α production with a similar behavior to that observed in LPS-induced tolerance (Fig. 2). No significant effect of CyP priming was shown on IL-6 production and gene expression in human monocytes; similarly, LPS-tolerant cells did not show any regulation of IL-6 expression (Fig. 2). The results differed from those obtained with THP-1 cells, and pointed out some differences between THP-1 and primary monocytes, having monocytes higher levels of IL-6 produced after LPS activation compared with THP-1 cells (Figs 1 and 2). Results about IL-1β in human monocytes showed a divergence between the protein released in cell culture supernatants and cytoplasmic mRNA expressions, highlighting the presence of tightly controlled regulatory mechanisms involving IL-1β secretion in primary monocytes. In detail, LPS-activated monocytes revealed negligible amounts of cytokine in cell culture supernatants even though high levels of mRNA coding for IL-1β were present in cell cytoplasm. CyP priming did not show any effect on IL-1β released in cell culture supernatants, and did not affect cytokine mRNA expressions compared with LPS-activated monocytes. The effects were completely different from those observed in LPS-tolerant monocytes, in which great amounts of IL-1β were found in cell culture supernatants and significantly higher mRNA expression were observed compared with LPS-activated cells (Fig. 2). Results about IL-10 expression did not show any significant effect of CyP priming both at the level of cytokine released in cell culture supernatants and at the level of mRNA expression (Fig. 2).

### CyP mediates down-regulation of TNF-α production by specifically inducing miR-146a and reducing IRAK-1 and TRAF6 expressions

**Figure 3:**
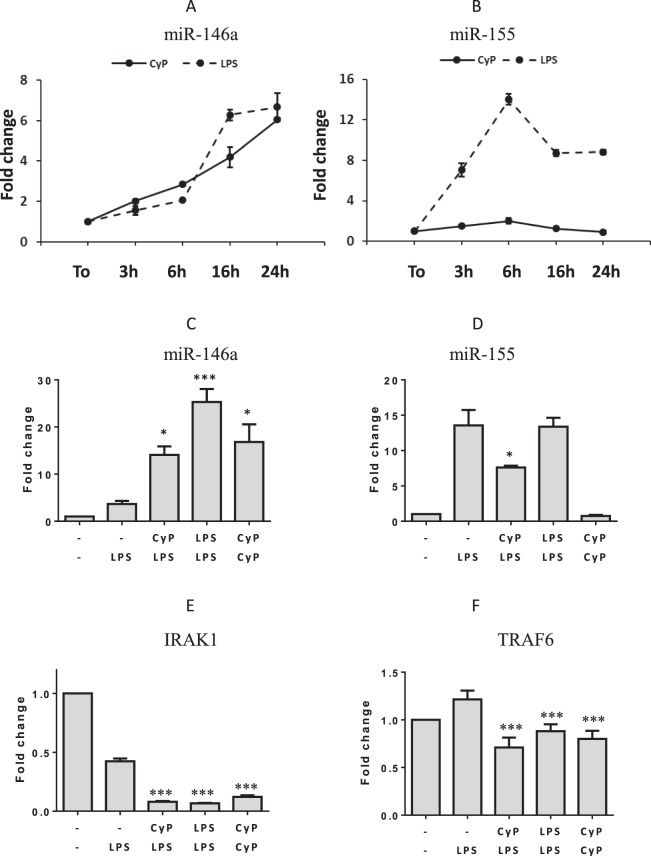
Effects of CyP on microRNA, IRAK1 and TRAF6 expressions. Time-dependent experiments of human monocytes incubated with CyP at the final concentration of 10 µg/ml and analysis of miR-146a (**A**) and miR-155 expression (**B**). Experiments with cells incubated with *E*. *coli* LPS (1 µg/ml) were performed as controls. The results are expressed as mean ± S.D. of triplicate data. Effects of CyP on miR-146a (**C**), miR-155 (**D**), IRAK1 (**E**) and TRAF6 (**F**) expressions in experiments of cross-tolerance with *E*. *coli* LPS. Data are presented as mean ± S.D. of triplicate data of one representative experiment of two performed. *p < 0,05; ***p < 0,001 (ANOVA with Tukey’s multiple comparison post hoc test).

It has been demonstrated that incubation of human THP-1 monocytic cells with *E*. *coli* LPS induces a dose- and time-dependent up-regulation of miR-146a and miR-155. Furthermore, miR-146a is specifically involved in TNF-α downregulation during LPS homogenous tolerance^15^. Since CyP is a LPS structure^24^, and is able to inhibit TNF-α production in experiments of cross-tolerance, we evaluated whether CyP treatment was able to affect miR-146a and miR-155 in human monocytes. Results showed that CyP increased miR-146a expression in a time-dependent manner, with a behavior similar to that observed in human monocytes stimulated with *E*. *coli* LPS (Fig. 3A). Differently from *E*. *coli* LPS, CyP did not show any induction of miR-155 (Fig. 3B). Further experiments were done to study the effects of CyP priming on miR-146a and miR-155 expression in experiments of cross-tolerance (Fig. 3C,D). Monocytes primed with CyP showed a significant increase of miR-146a expression in comparison with LPS-activated monocytes; the effects were similar to those observed in LPS-tolerant cells (Fig. 3C). Intriguingly, even monocytes incubated with two consecutive doses of CyP showed a significant increase of miR-146a expression (Fig. 3C). As shown in Fig. 3D, CyP priming inhibited miR-155 expression in experiments of cross-tolerance, while two consecutive administrations of CyP (CyP/CyP) did not affect miR-155 expression. Since miR146a is known to silence IRAK1 and TRAF6 adaptor proteins, affecting their mRNA stability, we also examined the expression of IRAK1 and TRAF6 in human monocytes primed with CyP or *E*. *coli* LPS. Results demonstrated that CyP pretreatment significantly inhibited both IRAK1 and TRAF6 expression in comparison with LPS-activated cells (IRAK1 mean inhibition 81%, TRAF6 mean inhibiton 41% CyP/LPS vs. −/LPS) (Fig. 3E,F). Results were similar to those obtained in homogeneous tolerance induction with *E*. *coli* LPS (Fig. 3E,F).

### CyP-induced miR-146a may account for cross-tolerance to LTA in THP-1 and human monocytes

**Figure 4:**
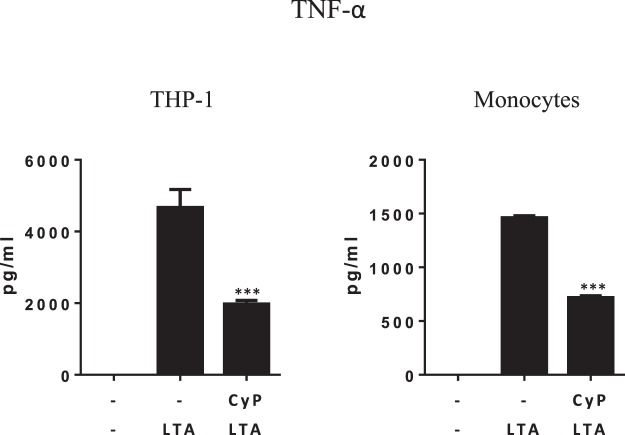
Reduction of TNF-α production by CyP in experiments of cross-tolerance to LTA. THP-1 cells and primary human monocytes were primed with CyP (10 µg/ml) for 16–18 h, washed twice, and then incubated with LTA from S. aureus (2 µg/ml) for 5 h. Supernatants were collected and TNF-α amounts measured by ELISA. Results are mean ± S.D. of triplicate cultures. ***p < 0,001 (t-test).

Since CyP was able to specifically induce, in a time-dependent manner, miR-146a in human monocytes, and upregulation of miR-146a downregulates TNF-α production inducing cross-tolerance to *E*. *coli* LPS, we wondered if CyP priming could affect the response to TLR2 ligands, such as LTA. CyP is a TLR4 antagonist and when incubated concomitantly with LTA does not inhibit TNF-α production. Indeed, priming of THP-1 cells and monocytes with CyP, significantly induced a state of hyporesponsiveness to subsequent incubation with LTA in terms of TNF-α production, as shown in Fig. 4, thus indicating miR-146a as a key mediator of CyP action.

## Discussion

Endotoxin tolerance represents a mechanism of regulation of the pro-inflammatory response following repeated LPS stimulations, having a relevant role in the control of the intensity and duration of innate immune activation. It has been recently demonstrated that epigenetic regulations, and particularly miR-146a, play a key role in the mechanisms of endotoxin tolerance and cross-tolerance, tightly controlling TNF-α production^10,15,22,25^.

In this study, we have analyzed the effects of CyP, a LPS obtained from *O*. *planktothrix FP1*, to assess whether CyP may be able to induce cross-tolerance to *E*. *coli* LPS. We employed CyP at the concentration of 10 µg/ml, a dose that was previously demonstrated to completely inhibit TNF-α production after co-incubation with *E*. *coli* LPS (1 µg/ml)^16,17^. Preliminary results in THP-1 monocytic cells demonstrated that CyP priming significantly inhibited TNF-α, IL-1β, IL-10 and stimulated IL-6 in experiments of cross-tolerance induction with *E*. *coli* LPS (Fig. 1). The effects were similar to those observed in homogenous LPS tolerance induction (Fig. 1). Further experiments performed to evaluate the effects of CyP priming in primary human monocytes only partially confirmed the data obtained on THP-1 cells (Fig. 2). CyP pretreatment showed a significant inhibition of TNF-α production and mRNA expression, in a similar manner to the effect observed in homogenous LPS tolerance, while the effects on IL-6, IL-1β and IL-10 did not confirm the results obtained in THP-1 cells (Fig. 2). Indeed, also the responses of human monocytes to LPS priming in homogenous tolerance induction resulted qualitatively different from those observed in THP-1 cells for IL-6, IL-1β and IL-10 (Fig. 2), suggesting caution when managing data obtained with cell lines. Although a general consensus has been established about the inhibitory effect of LPS re-exposure on TNF-α production in murine and human models^8,15,23,26–28^, the effects on other cytokines and chemokines are controversial^2,4,8,26,29–31^. Mengozzi and colleagues^5^ have clearly demonstrated that LPS-tolerant cells from healthy individuals differed in the regulation of IL-6, IL-8 and IL-1β productions, showing in some individuals inhibition and in others stimulation of these cytokines, whereas TNF-α resulted always inhibited. In our experiments, we also found out that a single optimal dose of LPS *in vitro* was enough to induce IL-1β mRNA, but not to induce the release of mature IL-1β in monocyte cultures. Only after twice-consecutive exposures to LPS, monocytes became active in the secretion of the mature protein. It has been demonstrated that IL-1β secretion is a highly regulated process in which proteins of the inflammasome network play a pivotal role^32,33^. Our results suggest that endotoxin homogenous tolerance can involve post-translational mechanisms for the fine regulation of IL-1β production. These mechanisms do not seem to be involved in CyP endotoxin cross-tolerance, since we did not find any induction of mRNA or IL-1β protein release by CyP priming.

Based on the results confirming a significant inhibition of TNF-α production by CyP priming both in THP-1 and human primary monocytes, we appraised whether the action of CyP was dependent on miRNA induction. Recent works have demonstrated that LPS interaction with TLR4-MD2 complex induces miRNA, mainly miR-146a and miR-155, which have been implicated in macrophage activation and endotoxin tolerance^25,34^. In particular, miR-146a induction was required for homogenous LPS tolerance in THP-1 monocytic cell line, having a critical role in the mechanism of suppression of TNF-α production (reviewed in^35^). In human monocytes stimulated with CyP alone, we found a significant and time-dependent increase of miR-146a expression, comparable to the induction produced by *E*. *coli* LPS stimulation. No effects by CyP treatment were shown on miR-155 expression, whereas there was a significant induction of miR-155 in cells treated with *E*. *coli* LPS alone (Fig. 3A,B). In cross-tolerance experiments, CyP priming considerably up-regulated miR-146a in comparison with LPS-activated cells, as observed in LPS-tolerant cells, thus suggesting a specific involvement of miR-146a in the mechanism of regulation of TNF-α production and tolerance induction by CyP. In particular, miR-146 up-regulation is inversely correlated with the expression of IRAK1 and TRAF6 adaptor proteins, which are direct targets of miR-146a^15,25^. In fact, CyP pretreatment induced a significant reduction of IRAK1 and TRAF6 expressions in human primary monocytes similarly to what observed during LPS homogenous tolerance (Fig. 3E,F). Interestingly, also control cultures employing two consecutive CyP administrations showed an increased miR-146a and reduced IRAK1 and TRAF6 expressions (Fig. 3C,E,F), whereas no effect on miR-155 expression was shown (Fig. 3D). These results suggest that CyP, a cyanobacterial LPS structure, can induce LPS cross-tolerance, by specifically inducing miR-146a. To confirm that the mechanism of action by CyP priming on TNF-α production could be mediated by miR-146a induction, we performed experiments on THP-1 cells and human monocytes, using CyP in the priming phase, followed by the TLR2 agonist LTA. When co-incubated with LTA, CyP was not able to inhibit the pro-inflammatory response induced by LTA as well as by other TLR2 ligands, such as peptidoglycans^16^. Indeed, a priming of 16–18 h with CyP was able to affect cell response to a subsequent incubation with LTA, by inhibiting TNF-α production both in THP-1 and human monocytes (Fig. 4). These results suggest that CyP can modify the innate immune response by affecting the levels of miR-146a expression. The mechanisms behind CyP influence on miR-146a are currently unknown. Previous studies have shown that CyP does not induce NF-κB activation in cells transfected with a NF-κB-driven luciferase reporter together with an expression vector encoding TLR4^16,17^. Furthermore, CyP also inhibited spontaneous luciferase activity of TLR4 transfectants, measured in the absence of LPS^16^. These results suggest that the mechanism by which CyP induces miR-146a expression, is likely NF-κB-independent, although more experiments are needed to fully clarify this point.

CyP structure has been recently elucidated; it differs from typical *E*. *coli* LPS for the presence of two to four acyl chains in the lipid A moiety, it is devoid of phosphate groups and carries galacturonic acids^19,24^. Recently, it has been demonstrated that LPS molecules from Bacterioides of gut microbiota, such as *B*. *dorei*, with a monophosphorylated lipid A carrying four and five acyl chains failed to induce any pro-inflammatory response^36^. However, differently from CyP, LPS from *B*. *dorei* failed to induce protective endotoxin cross-tolerance^36^. No data are reported about possible effects of LPS from Bacteriodes on miRNA induction. Data about LPS from other gram-negative bacteria, as *Phorphyromonas*, *Helicobacter* and *Salmonella* strains^37–39^, showed both miR-146a and miR-155 inductions, as observed for *E*. *coli* LPS, suggesting that miRNAs regulations represent a common post-transcriptional way to regulate the immune response. At present, we do not know any LPS, except CyP, specifically affecting miR-146a. This specific action, associated to the absence of any induction of a pro-inflammatory response by CyP, could be of great relevance for therapeutic intervention, as demonstrated *in vivo* in experimental models of epilepsy^40^, and it highlights the importance of epigenetic regulations to influence the disease course in some pathological conditions.

## Material and Methods

### Cells and reagents

Human THP-1 monocytic cell line was obtained from Istituto Zooprofilattico Sperimentale della Lombardia e dell’Emilia (Brescia, Italy). Cells were maintained by twice weekly passage in complete RPMI 1640 medium (Gibco, Grand Island, NY), containing 10% fetal bovine serum (FBS, Gibco, Grand Island, NY), 2mM L-glutamine (Euroclone, Milano, IT), 50 U/ml penicillin, 50 ug/ml streptomycin (Euroclone, Milano, IT), 0.05 mM 2-mercaptoethanol. Cells were incubated at 37 °C in 5% CO_2_ and cultures were performed at a density of 5 × 10^5^/ml in all experiments.

Blood was obtained from healthy donors after informed consent by Transfusional Service of Ospedale di Circolo Fondazione Macchi (Varese, IT), in accordance with relevant national guidelines and laws (D.L. n. 219, October 21, 2005; D.L. suppl. 69 G.U. n. 300, December 28, 2015). Monocytes were isolated from buffy coats by density gradient centrifugation on Lymphoprep (Axis-Shield PoC AS, Oslo, N) and purified from peripheral blood mononuclear cells by positive selection using anti-CD14–conjugated magnetic microbeads (Miltenyi Biotec Bisley, UK). Cells were incubated at 37 °C in 5% CO_2_ in complete RPMI medium and employed in culture at a density of 5 × 10^5^/ml.

Cyanobacterial LPS antagonist (CyP) was extracted from the freshwater *cyanobacterium Oscillatoria planktothrix FP1* by a phenol-guanidinium thiocyanate-based method, as described elsewhere^16,40,41^. The product was treated with DNase (20 µg/ml) and RNase (10 µg/ml) in 50 mM TRIS buffer, pH 7.5 containing 10 mM MgCl_2_, for 2 h at room temperature prior to addition of proteinase K (100 µg/ml) for an overnight incubation at 37 °C. The sample was then re-extracted, purified by ion exchange chromatography with Sartobind Q (Sartorius Stedim Biotech, Goettingen, D), centrifuged in Zeba spin desalting columns (Pierce, Rockford, IL) and freeze-dried. The final product was analyzed by size exclusion chromatography in HPLC, using evaporative light scattering detector (column Superose 12; GE healthcare Little Chalfont, UK; HPLC, Agilent 1200), visualized by electrophoresis in SDS-PAGE and silver staining after periodate oxidation, or by staining with Pro-Q Emerald 300 lipopolysaccharide gel stain kit (Invitrogen Carlsbad, CA)(Supplementary Fig. S1). Purity of CyP was >90%. Protein contamination was <2% (Bradford method); nucleic acid contamination measured spectrophotometrically (260–280 nm) was 0.5%. CyP preparations contained <1 Endotoxin units/µg measured by endpoint chromogenic LAL test (Lonza Group, Basel, CH). CyP was dissolved in sterile, endotoxin-free phosphate-buffered saline (PBS) prior to addition to cell cultures.

Ultrapure LPS (from *E*.*coli* serotype O111:B4) was purchased by Sigma Aldrich (St. Louis, MO), lipoteichoic acid (LTA from *S*. *aureus*) was purchased by Invivogen (San Diego, CA 92121) and dissolved in sterile endotoxin free PBS at a concentration of 1 mg/ml and 2.5 mg/ml, respectively. Further dilutions were made in complete medium.

### *In vitro* induction of cross-tolerance by CyP

Tolerance and cross-tolerance inductions were performed according to the methods described by Nahid *et al*.^15,22^ with some modifications. Cells at the concentration of 5 × 10^5^ cell/ml were seeded in sterile 6-well plates, and pre-incubated with CyP, at the final concentration of 10 µg/ml for 16–18 h at 37 °C in 5% CO_2_. Control cultures consisted of cells pre-treated in medium alone, and cultures pre-incubated with *E*. *coli* LPS (1 µg/ml) for homogeneous tolerance induction. After incubation, cells were harvested, washed twice with PBS and resuspended in fresh complete medium with or without LPS (1 µg/ml), or CyP (10 µg/ml). Cells were maintained at 37 °C in 5% CO_2_ for 5 h, then supernatants and cell pellets were collected and stored at −80 °C until cytokine analyses and RNA extractions were done. The experiment consisted of five treatments: unstimulated control (−/−), single dose LPS stimulated cells (−/LPS), CyP pre-treatment followed by LPS (CyP/LPS cross-tolerance), LPS pre-treatment followed by the same dose of LPS (LPS/LPS, endotoxin tolerance), CyP pre-treatment followed by the same dose of CyP, as further control (CyP/CyP). Other experiments were done using CyP pre-treatment (10 µg/ml) followed by LTA at the final concentration of 2 µg/ml (CyP/LTA cross-tolerance). At the end of all incubations, cell viability was assessed by trypan blue exclusion test.

### Cytokine ELISA

TNF-α, IL-6, IL-1β, IL-10 were measured in supernatants of cell cultures using enzyme-linked immunosorbent assays (ELISA) kits by eBioscience Affymetrix (S. Diego CA), according to manufacturer’s instructions, and calibrated with commercial cytokine molecules in the kits. Absorbance was measured at 450 nm with TECAN microplate reader (Tecan Group Ltd, Mannedorf, CH); standard curves were constructed and cytokine concentrations of the samples were read off these curves.

### RNA isolation

RNA isolation was carried out using automated Maxwell instrument and Maxwell 16 LEV simplyRNA cell kit (Promega, Corporation, Madison, WI). The RNA concentration was determined using a NanoDrop spectrophotometer and equal amounts of each RNA were used for real-time PCR (qRT-PCR) analysis.

### Quantitative real-time PCR for cytokine and IRAK1, TRAF6 adaptor protein expressions

Expression of mRNAs was analyzed using Iscript cDNA synthesis kit and ITAQ Universal Probes Supermix (Bio-Rad Laboratories s.r.l., Milano, IT). Briefly, 400 ng total RNA was reverse transcribed in a final volume of 20 µl, according to manufacturer’s instructions. PCR was conducted in a 20-µl volume reaction containing 4 µl 1/5 diluted cDNA template, and PrimePCR^TM^ probe assays (TNF-α unique assay ID qHsaCEP0040184; IL-6 qHsaCEP0051939; IL-1β qHsaCIP033362; IL-10 qHsaCEP0051966; IRAK1 qHsaCEP0057865; TRAF6 qHsaCIP0028638) labeled with a FAM fluorophore (Bio-Rad Laboratories s.r.l., Milano, IT). Fold changes were calculated compared with unstimulated controls (−/−), after normalizing the change in expression of the gene of interest to the housekeeping gene β-actin (unique assay ID qHsaCEP0036280), using the ΔΔCt method^42^.

### MiRNA quantitative real-time PCR

MiRNA analyses were performed using miRCURY LNA^TM^ Universal RT microRNA PCR kit (Exiqon, A/S, Vedbaek, DK). Briefly, 50 ng of RNA were reverse transcribed in 10 µl final volume, according to manufacturer’s instructions. Quantitative real-time PCRs were performed in final volumes of 10 µl reaction containing 4 µl 1/25 diluted cDNA template, using SYBR green and LNA^TM^ enhanced primers (targets: hsa-miR-146a-5p, hsa-miR-155–5p) designed by Exiqon for optimal performance with miRCURY LNA^TM^ Universal cDNA Synthesis kit II and ExiLENT SYBR. Melting curves were done to confirm that any product was specific to the desired amplicon. Stably expressed miR-103a-3p was employed as reference gene and used for normalizing target expressions. RNA spike-ins and the matching primer pairs, included in the kit, were employed in some experiments as controls of the RNA isolation, the cDNA synthesis reaction and the PCR.

### Statistical analysis

One-way ANOVA with Tukey’s Multiple Comparison post hoc test and t-student test were performed to assess statistical significance (P values < 0.05) using GraphPad Prism 6.0 (GraphPad software).

## Electronic supplementary material

Supplementary Material
