# Supplementary material for: MiR-146a induction by cyanobacterial lipopolysaccharide antagonist (CyP) mediates endotoxin cross-tolerance
Source: Sci Rep. 2018 Jul 27;8:11367. doi: 10.1038/s41598-018-29820-w (PMC6063882; doi:10.1038/s41598-018-29820-w)

MiR-146a induction by cyanobacterial lipopolysaccharide antagonist (CyP) mediates endotoxin cross-tolerance

Monica MOLTENI, Annalisa BOSI, Vincenzo SATURNI, Carlo ROSSETTI

Figure S1.

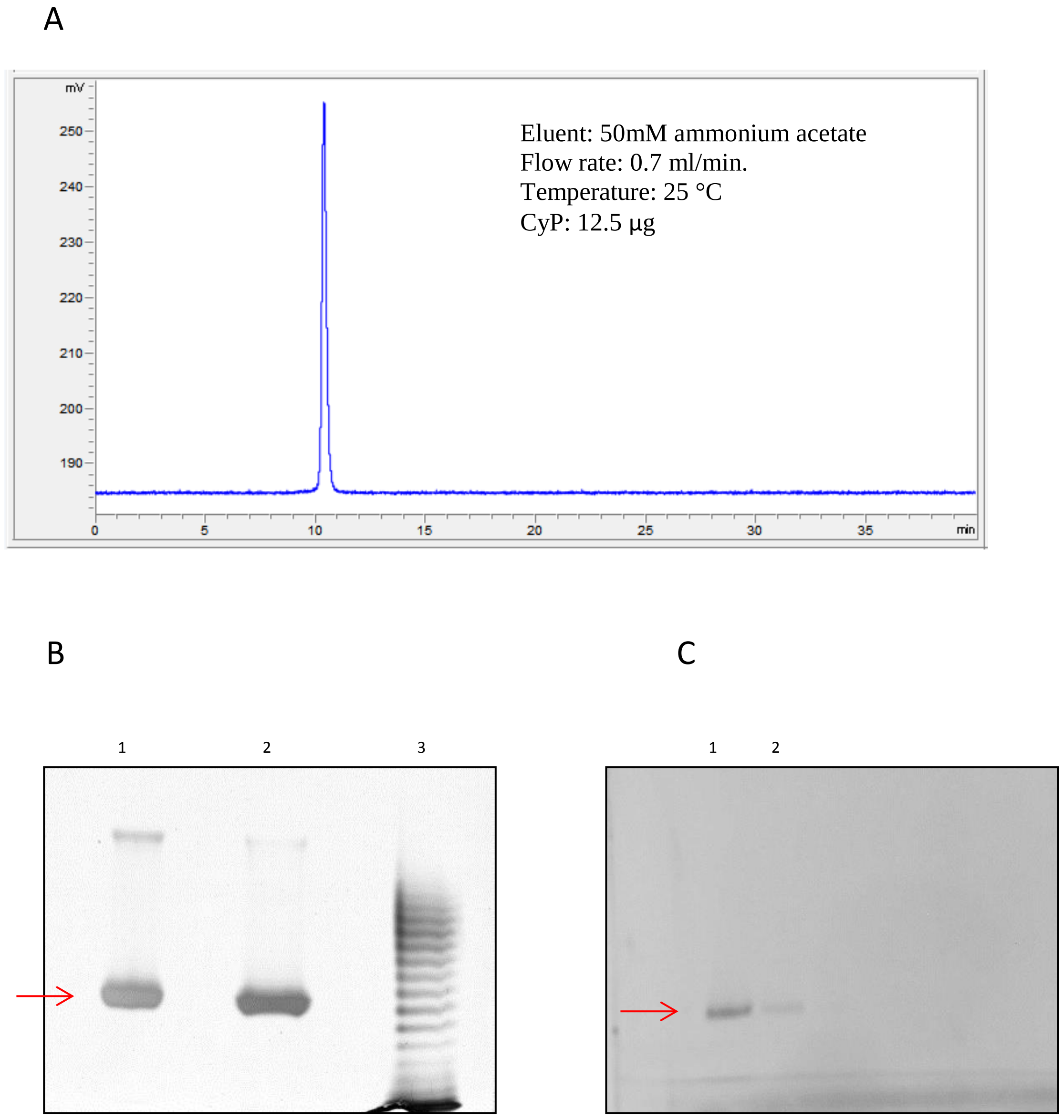

Lane 1: CyP 5 µg
Lane 2: CyP 5 µg (in solution with 1 mM EDTA)
Lane 3: E. coli LPS (serotype O111:B4) 8 µg

Lane 1: CyP 2 µg
Lane 2: CyP 0,5 µg

MiR-146a induction by cyanobacterial lipopolysaccharide antagonist (CyP) mediates endotoxin cross-tolerance

Monica MOLTENI, Annalisa BOSI, Vincenzo SATURNI, Carlo ROSSETTI

Figure S2.

A

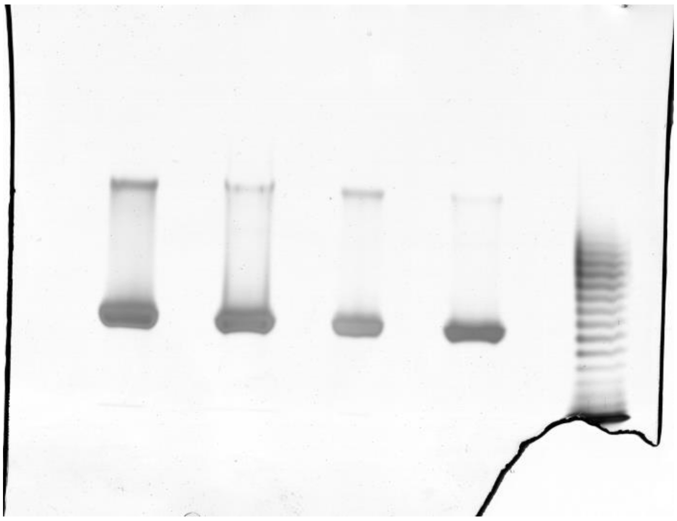

B

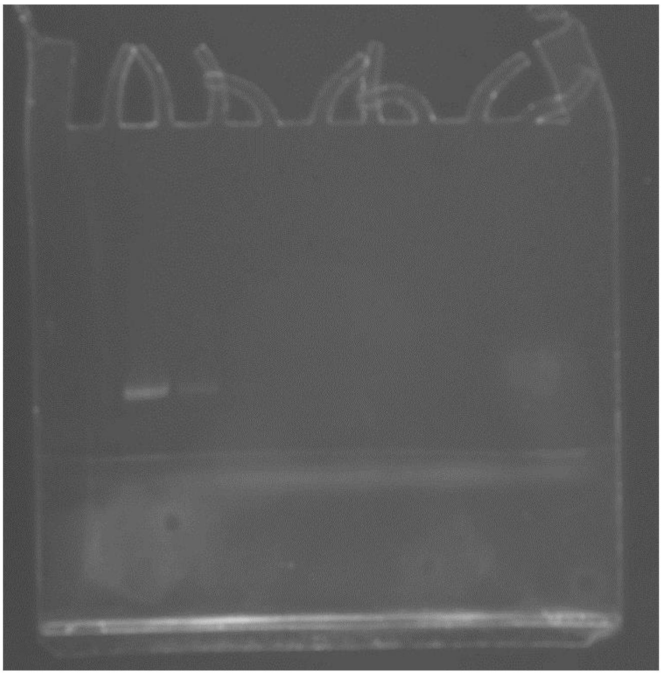

Supplement: Supplementary file 1 — Supplementary Material [file 41598_2018_29820_MOESM1_ESM.pdf]
